# Editorial: Untangling the Role of Tau in Physiology and Pathology

**DOI:** 10.3389/fnagi.2020.00146

**Published:** 2020-05-26

**Authors:** Miguel Medina, Jesús Avila

**Affiliations:** ^1^Centro de Investigación Biomédica en Red sobre Enfermedades Neurodegenerativas, ISCIII, Madrid, Spain; ^2^CIEN Foundation, ISCIII, Queen Sofia Foundation Alzheimer Center, Madrid, Spain; ^3^Centro de Biología Molecular Severo Ochoa, CSIC-UAM, Madrid, Spain

**Keywords:** Alzheimer, dementia, microtubules, neurodegeneration, tau, tauopathies

We are pleased to present a Topic Research and e-book specifically devoted to the role played by tau protein both in physiology and pathology. The recognition of tau as a key player in the pathobiology of human neurodegenerative diseases has driven substantial efforts to understand its biological and pathological function. This issue includes a series of original papers and updated reviews covering recent advances on a variety of aspects of tau biology (protein structure, genetics, gene expression regulation) and its role in human neurodegenerative disorders (pathology spreading, novel therapeutic approaches) as well as its potential role in other physiological and pathological processes.

Under physiological conditions, tau is expressed throughout the brain and acts as a microtubule-associated protein (MAP) by directly binding to microtubules and dynamically regulating its structure and function. Tau is primarily a neuronal protein encoded by a single gene that in the CNS can result in six major isoforms by alternative splicing. Beyond its classical role as a MAP, recent advances in our understanding of tau cellular functions have revealed novel insights into its function on various cellular processes.

Under pathological conditions though, tau detach from microtubules and self-assembles to form insoluble intracellular filaments that constitute a common pathological feature of various human neurodegenerative disorders collectively known as tauopathies, including frontotemporal lobar degeneration (FTLD), progressive supranuclear palsy (PSP), and Alzheimer's disease (AD), the latter being the most common of them. More than three decades have passed since the seminal discovery that identified hyperphosphorylated tau protein as the main component of the paired helical filaments (PHF) present in the neurofibrillary tangles (NFT) isolated from the brain of Alzheimer's disease (AD) patients. Since then, evidence has accumulated showing that regulation of tau behavior and function under physiological and pathological conditions is mainly achieved through post-translational modifications (PTM), including phosphorylation, glycosylation, acetylation, and truncation among others, indicating the complexity and variability of factors influencing regulation of tau toxicity, all of which have significant implications for the development of novel therapeutic approaches in various neurodegenerative disorders. The tau-driven neurodegenerative process is most likely due to a combination of changes in post-translational modifications and alterations in protein folding and clearance.

In this Research Topic, Barbier et al. reviews the many advances have been made in the understanding of tau function as a MAP, and in the structural aspects of its interaction with microtubules (MTs). The role of the different regions on tau protein and various post-translational modification in the tau/MTs interaction is reviewed in detail. The article by Trushina et al. focuses on phosphorylation, the most studied tau PTM using a bioinformatics analysis to study changes in phosphorylation sites during evolution at different regions on the tau molecule that could determine novel protein interactions and has potential implications in the development of human tauopathies. The article by Hernández et al. then addresses differences in the aminoacid sequence between human and murine tau, in particular at the N-terminal where a short human-specific 12-residues domain may account for differences in interacting partners and secretion patterns between both species, which could ultimately affect tau pathology spreading mechanisms.

The occurrence of filamentous structures of aggregated, hyperphosphorylated tau is a constant finding in tauopathies, generally associated with synaptic loss and neuronal death. These are clinically, morphologically, and biochemically heterogeneous disorders characterized by the deposition of abnormal tau protein in the brain. They are differentiated by distinct neuropathological phenotypes based on the involvement of different anatomical areas, cell types, and presence of distinct isoforms of tau in the pathological deposits. The precise mechanisms for the initiation of tau fibrillization and aggregation are addressed by Fichou et al. which focuses on the role of cofactors in the formation of low-order complexes that will then favor seeding of higher order irreversible species. The process from physiological tau soluble conformation toward pathologicas aggregated forms is also addressed by Vega et al., in this case by studying the role of the calcium-binding protein EFhd2 in the structural dynamics of tau proteins.

Long deemed as an axonal protein in mature neurons, tau also accumulates in the somatodendritic compartment and in the synaptic compartment in healthy brains, suggesting a role for tau in regulating normal synaptic function. In this context, the article by Kobayashi et al. addresses the role of tau at the synapse and show that glutamate transiently stimulate tau mRNA translation in a dose-dependent manner in neuroblastoma cells and discuss its potential implications of synaptic tau in neurodegeneration.

While no mutations in the MAPT gene have been described to cause familial AD, rare, dominant mutations cause an inherited form of frontotemporal dementia and parkinsonism. Furthermore, the MAPT H1 haplotype is strongly associated as a risk factor for AD and other tauopathies. This topic is further addressed in the article by Sánchez-Juan et al. in which they study two large Spanish cohorts and show that genetic variants linked to the MAPT H1 haplotype constitute genuine risk alleles for AD, particularly in APOE-ε4 non-carriers.

Disturbances in circadian rhythms and disruption of the sleep-wake cycle are common symptoms of AD and often occur early in the course of the disease process. Consequently, Arnes et al. address the potential mechanisms underlying the role of tau in sleep pattern regulation by using *Drosophila* as an animal model and suggest that it is mediated through modulation of cytoskeleton dynamics in terminal projections of the circadian pacemaker neurons.

During the last decade emerging experimental evidence suggest that tau pathology spreads throughout the brain in a stereotypical manner that reflects the propagation of abnormal tau species along neuroanatomically connected brain areas. The proposed prion-like mechanism involves the transfer of abnormal tau seeds from cell to cell and recruitment of normal tau in the recipient cell to generate new tau seeds, thus leading to propagation of pathology. The article by Karikari et al. tackle this issue by studying the effect of FTD mutations in the aggregation, conformation, and cellular uptake of various tau species and fragments. Next, Smolek et al. describe how genetic background may have a significant effect on tau pathology spreading in murine models. They proposed the immune response modifying the genetic background as a factor influencing the susceptibility to, and propagation of, tau pathology.

This later point relates with a group of three contributions that deal with the role of non-neuronal cells in the pathogenesis of AD and other tauopathies. First, the article by Španić et al. reviews the role of microglia in the spreading of tau pathology, in particular the involvement of exosome synthesis and inflammasome activation, suggesting that tau seeding and microglial activation could be a key element in development and progression of the neurodegenerative process. Second, Ferrer et al. present data from studies using human brain homogenates from various human tauopathies into the corpus callosum of wild type mice and show the occurrence of tau seeding in oligodendrocytes and tau spreading, suggesting the involvement of the white matter as a pathogenic component in tauopathies. The complex interaction between tau and astroglia is the focus of a thorough review article by Kovacs in which biochemical, neuropathological, and pathogenic aspects with relevance to astroglia are discussed together with a extensive morphological description of the various tau-bearing astrocytes found in the in human tauopathies.

Development of rational tau-based drug therapies calls for a deeper understanding of the role of post-translational modifications in regulating tau pathways leading to dysfunction and neurodegeneration. Thus, for instance, Higham, Malik et al. identifies ankyrin 1, previously identified through epigenomic analysis to be associated with AD, as a novel potential therapeutic target for AD based on *in vivo* studies in *Drosophila*. In an accompanying article, the same group (Higham, Hidalgo et al.) present data on the role for L-type calcium channels in restoring olfactory memory in tau transgenic flies and discuss whether targeting them may have therapeutic potential.

Ivashko-Pachima and Gozes review evidence showing the *in vitro* neuroprotective activity of a short peptide that works through a group of MT plus-end tracking proteins known as End Binding proteins, proposing the future development of drug candidates containing that peptide sequence. Furthermore, Siano et al. present *in vitro* data to support the use of a known inhibitor of the ERK pathway currently in clinical development for various tumors as a drug candidate for AD based on its ability to inhibit tau hyperphosphorylation and aggregation in cell-based assays. Lin et al. then go step further to describe data of a proof-of-concept study in mouse AD models after chronic treatment with an antibody against programmed death cell protein-1 (PD-1). Their results show a modest improvement in the motor phenotype, but no significant effect on learning and memory tasks nor reduced tau pathology.

The last three contribution to this Research Topic deal with the role of tau in other pathologies. Thus, Fernández-Nogales and Lucas review emerging evidence showing tau pathology in Huntington's disease (HD), from increased total tau levels, alteration in alternative splicing, hyperphosphorylation, or truncation to the presence of cytoplasmic aggregates and nuclear envelope invaginations. On the other hand, Alves et al. present data showing tau hyperphosphorylation in epileptic mouse models and discuss the potential implications of multiple convergent mechanisms in AD and epilepsy. Finally, Gargini et al. review recent evidence on the expression and role of tau in cancer, especially in brain tumors such as gliomas, that mat uncover novel aspects of tau biology.

In summary, all this increased understanding of the molecular mechanisms involved in tau function and dysfunction allow us to define a detailed blueprint of the tau cellular functional network, certainly providing new clues into its role in physiology as well as to design more efficient therapeutic approaches to tackle human tauopathies. This Research Topic intends to provide an overview of our current knowledge on the role of tau protein in the nervous system under physiological conditions as well as in human pathologies such as neurodegenerative tauopathies. Classical and atypical functions as well as its involvement in the propagation of tau pathology in tauopathies and other pathologies are also discussed.

## Author Contributions

MM and JA have participated in writing the editorial and both have acted as Topic Editors.

## Conflict of Interest

The authors declare that the research was conducted in the absence of any commercial or financial relationships that could be construed as a potential conflict of interest.

